# Final-year students’ perceptions of online integrated primary care learning

**DOI:** 10.4102/safp.v67i1.6034

**Published:** 2025-01-21

**Authors:** Aviva Ruch, Joel Francis, Ann Z. George

**Affiliations:** 1 Unit of Undergraduate Medical Education, School of Clinical Medicine, Faculty of Health Sciences, University of the Witwatersrand, Johannesburg, South Africa; 2 Department of Family Medicine and Primary Care, School of Clinical Medicine, Faculty of Health Sciences, University of the Witwatersrand, Johannesburg, South Africa; 3 Centre for Health Science Education, Faculty of Health Sciences, University of the Witwatersrand, Johannesburg, South Africa

**Keywords:** clinical teaching, online learning, medical students, medical education, COVID-19, decentralised training, integrated primary care

## Abstract

**Background:**

Integrated primary care (IPC) is a final-year medical subject at the University of the Witwatersrand, Johannesburg, South Africa. It focusses on primary health care training. The coronavirus disease 2019 (COVID-19) pandemic exacerbated existing decentralised training challenges, including standardisation and patient exposure. This study explored IPC students’ experiences and perceptions of online learning during the COVID-19 pandemic.

**Methods:**

This explanatory-sequential mixed-methods study was informed by the technology acceptance model, community of inquiry model and self-regulated learning theory. A cross-sectional online survey was followed by focus group discussions (FGDs) (*n* = 2 and *n* = 3, respectively). All 316 medical students in the 2021 cohort were eligible to participate. Closed-ended survey responses were analysed using descriptive and inferential statistics. Open-ended responses were analysed using content analysis. The FGDs were thematically analysed.

**Results:**

The survey response rate was 52% (*n* = 164/316). Most students found the online content easily accessible (93.3%) and logically organised (80.0%). The course structure and organisation, and the range of online activities offered were the main features that supported learning. The main challenges included the content not being comprehensive and the difficulty of learning patient management from online content. Suggested improvements related to the course design and ways students and instructors can maximise the affordances of the online course.

**Conclusion:**

Acknowledging the limitations of learning clinical content online, the participants felt the course supported their learning. Our findings suggest that well-designed online content can augment clinical learning.

**Contribution:**

This study contributes to the discourse on the value of online learning for clinical teaching.

## Background

Family medicine is a fundamental discipline in the South African medical curriculum, given the desperate need to shift to a generalist approach to medicine^1^ and to address the inequitable distribution of health professionals and resources.^2^ However, training healthcare professionals to meet the country’s needs is challenging in the face of under-resourced teaching facilities, under-staffed facilities with overworked health professionals, hospital budget cuts and a human immunodeficiency virus (HIV) epidemic.^1^ The current doctor-to-patient ratio in South Africa is 1:3198, a decline from the 2019 ratio of 1:1266, and is far lower than other low- to middle-income countries (LMICs),^3^ posing a further challenge.

The University of the Witwatersrand (Wits University) is unique in South Africa in that it allows graduates to join the third year of the 6-year Bachelor of Medicine, Bachelor of Surgery (MBBCh) programme. Thereafter, the programme is known as the Graduate Entry Medical Programme (GEMP). Final-year medical students are thus in GEMP IV. The GEMP IV is divided into eight teaching blocks. The 6-week Family Medicine block is called Integrated Primary Care (IPC) because it covers common presenting conditions from Internal Medicine, Surgery, Psychiatry, Obstetrics and Gynaecology, Family Medicine, Community Paediatrics and Public Health, and focusses on the management of these conditions at a primary health care (PHC) level. The IPC block offers undergraduate students their first experience of PHC. Other South African universities similarly emphasise PHC exposure at the undergraduate level. For example, Stellenbosch University’s final-year integrated longitudinal clerkship for medical students provides exposure to PHC.^4^

Decentralised training (DCT) differs from traditional training in that it occurs across a wide geographical area instead of large central tertiary teaching hospitals.^5^ Integrated primary care is taught at 12 DCT sites affiliated with Wits University across three of South Africa’s nine provinces. Each province is divided into health districts where DCT is conducted at PHC clinics, community health centres or district hospitals. Evaluations of DCT, predominantly in Australia^6^ and North America but increasingly in South Africa and other African countries,^5,7,8^ have found that DCT poses challenges around standardising teaching and patient interaction across sites. However, in addition to exposing students to PHC, DCT fosters competency in holistic patient care at a primary level of care, rural medicine, working in under-resourced facilities^8^ and addresses global shortages of medical doctors in rural areas and student congestion.^7^

Online learning is essential for successful DCT and it promotes standardisation and access to learning resources.^5^ In Botswana, online learning has helped address faculty shortages across DCT sites and has encouraged collaboration and communication between learners, experts and peers.^9^ In South Africa, researchers from several universities recognised information and communication technology (ICT) as a tool to assist medical students in developing common core competencies and, more particularly, address the challenges of training medical students at sites away from the central university teaching platform.^5^

The coronavirus disease 2019 (COVID-19) pandemic necessitated drastic changes to medical education worldwide^10^ and exacerbated existing challenges of training final-year medical students through, for example, decreased student–patient interaction.^11^ Numerous research studies on the emergency transition to online learning during the pandemic provided the education community with insights into student perceptions and experiences.^12,13,14^ In developing countries such as Pakistan, students in higher-education institutions faced numerous challenges with using and accessing online learning and thus found online learning less desirable than traditional classes.^12^ Some of Pakistan’s higher-education students said they would not like to continue with online learning after the pandemic.^13^ A comparative study of online learning in South Africa, Wales and Hungary during the pandemic found university students’ attitudes towards online learning differed between those three countries.^14^ South African and Welsh university students preferred face-to-face teaching and found it challenging to engage online, but most noted that their learning environment and the design of the learning content contributed to their attitude towards learning online and their ability to engage with online content.^14^

The potential for ICT to enhance teaching and learning and to offer a broader range of learning opportunities to a wider range of students^15^ has long been recognised by governments and universities.^16^ However, the uptake of ICT in education depends on institutional readiness, which is often not optimal in low- to middle-income countries (LMICs),^17^ and whether educators use the technologies effectively. In medical education, the uptake of online learning has varied across and within medical schools, and has been slower than adoption across other higher-education faculties.^18^ Possible reasons for the slow uptake may be the lack of a single medical-education context and the fact that different medical disciplines train students in various settings according to the domain being taught and the nature, content, context and learning objectives of the learning event.^19^ There are also limits to how online training can be used in medical education. For example, online content can only augment clinical training but cannot replace bedside teaching for senior clinical students.^20^ Some authors suggest that ICT may assist with achieving many teaching and learning objectives well beyond the pandemic,^11^ making exploring students’ experiences and perceptions of online learning in the post-pandemic period imperative. Such knowledge could facilitate strategically combining online and face-to-face learning in what is known as ‘blended learning’.^21^ Blended learning is an alternative to fully online learning; the latter lacks the face-to-face or in-person component^21^ integral to blended learning.

The aim of this study was to explore final-year medical students’ experiences and perceptions of online learning across DCT sites during the COVID-19 pandemic at Wits University in 2021.

### Theoretical underpinnings of the study

The investigation of students’ perceptions and experiences of IPC online learning was informed by the technology acceptance model (TAM),^22^ the community of inquiry (CoI) model^23^ and self-regulated learning (SRL) theory.^24^ The TAM has undergone numerous iterations^25^; however, the original model is still widely used to determine the acceptability of new technology and its intended use.^25,26^ The TAM focusses on perceived usefulness and ease of use, which directly affect user acceptance of technology and is therefore relevant to this work.^26^

Like the TAM, the CoI model has been used extensively in research into online and blended-learning contexts.^18,23^ The CoI model nurtures a collaborative, constructivist learning approach by focussing on the interaction of three core elements: cognitive presence, social presence and teaching presence.^23^ Cognitive presence refers to ‘the extent to which learners can construct and confirm meaning through sustained reflection and discourse in a critical community of inquiry’.^23^ In short, cognitive presence involves the students’ ability to think critically and construct meaningful knowledge.^27^ Social presence refers to students’ ability to present themselves and foster relationships in the learning community.^23^ The social presence supports the cognitive presence by facilitating interaction and fostering critical thinking around learning topics and experiences.^27^ Teaching presence refers to the educator’s role in content selection, course and content organisation, the delivery of the course material and the design and development of assessment activities.^23^ Teachers are also responsible for fostering a safe and engaging learning environment to enhance social presence.^27^ The three forms of presence comprising the CoI do not exist in isolation but are essential to ensuring high-quality educational experiences.^27^

Self-regulation forms the basis for the action with purpose that motivates learner performance.^28^ Vancouver,^29^ citing DeShon and Rench (2009) and Zeidner, Boekaerts and Pintrich (2000), explained that self-regulation is a meta-theory about motivation and satisfaction with one’s achievements. Metacognition supports the acquisition and retention of knowledge and skills in SRL.^30^ The SRL meta-theory also potentially encourages students to be lifelong learners who will organise their time and learning^31^ and self-monitor their progress,^32^ thereby creating a more effective learning experience. Moos and Ringdal^33^ suggest that online learning, where teachers plan learning events to foster student participation, promotes SRL and that SRL is enhanced by the teacher, cognitive and social presence described in the CoI model.

## Research methods and design

### Study design

This explanatory-sequential mixed-methods study consisted of an online survey and focus group discussions (FGDs). The survey findings informed the questions used in the FGDs.

### Data collection

#### Online survey

All 316 final-year medical students in 2021 who had completed the IPC rotation were eligible to participate in the study. An estimated required sample size of 176 students was calculated using a confidence interval (CI) of 95% with a 5% margin of error, which required a response rate of 54% from the students.

The online questionnaire, consisting of 21 closed-ended and 4 open-ended questions, was developed in REDCap. Section 1 of the questionnaire, adapted from the original TAM questionnaire,^34^ explored the perceived usefulness and ease of use of the online content and respondents’ attitudes to and acceptance of online learning. The section that followed (16 questions) was underpinned by the CoI model. The questions asked about the relevance and application of the online content, whether the content and learning interactions augmented their clinical skills, and their preferred learning formats (e.g., videos and quizzes). The final section asked about gender, age and racial classification. Race was included as a demographic category to investigate the representativeness of the sample versus the GEMP IV cohort. The racial classifications used were those introduced during the apartheid era (1947–1994) according to the *South African Population Registration Act* (No. 30 of 1950). These classifications are still used to assist the South African government in redressing injustices against previously disadvantaged population groups. The questionnaire ended with a request for students interested in participating in the subsequent FGDs to provide their email addresses.

The survey link was sent on 11 November 2021, with weekly reminders until the survey was closed on 30 November 2021. Internet protocol (IP) addresses were used to prevent duplicate entries. The first page of the questionnaire provided information about the study. Once students provided informed consent, they could complete the questionnaire. The open-ended questions contributed to the ‘preliminary understanding’^35^ of phenomena for further probing in the FGD. A pilot study conducted in early November 2021 with 10 volunteers from the 2020 final-year cohort was used to improve the readability and clarity of the questions.

#### Focus group discussions

Twenty-seven volunteers were emailed participant information sheets and consent forms. However, only five participants arrived for the focus groups. Two FGDs (*n* = 2; *n* = 3, respectively) were conducted in December 2021 using Microsoft Teams. Creswell and Plano Clark (pp. 190–191)^36^ recommend that, in explanatory-sequential mixed-method studies, ‘the qualitative data collection comes from a much smaller sample than the initial quantitative data collection’. These authors also emphasise that it is not the number of participants but analysing and interpreting the data to provide adequate explanations for selected results from the quantitative phase that is paramount in explanatory-sequential mixed-method studies.^36^ Based on our survey findings, the FGD participants were asked what activities best supported their learning, what challenges they experienced with the course, their experience of the online tutorials, whether the online content and interactions prepared them to manage patients and what they consider the students’ role in the learning process.

### Data analysis

The closed-ended data were analysed in Stata 15. Inferential techniques included the use of correlations and associations tested using chi-squared tests. Fisher’s exact test was used where expected counts were less than 5%. All tests were conducted at a significance level of *p* ≤ 0.05. The Likert-scale categories ‘agree’ and ‘strongly agree’ and ‘disagree’ and ‘strongly disagree’ were aggregated and are reported as ‘agree’ and ‘disagree’. The open-ended answers were transferred to Microsoft Excel for content analysis, which allows data to be analysed qualitatively and quantified.^37^ Content analysis uses a descriptive approach for coding and interpreting the frequency counts for the categories and sub-categories derived from the coding process.^37^ We report the frequency counts graphically, with the categories as legends to facilitate understanding of the sub-category counts represented by the bars in the graphs. The major results from the content analysis of the online questionnaire informed the FGD questions.

The de-identified verbatim transcripts from the focus group sessions were analysed thematically^38^ using MAXQDA 2022. The patterns identified from coding the focus group transcripts were grouped into themes and sub-themes and mapped to display the relationships between them.^38^ The thematic analysis is thus reported as thematic maps with frequency counts to show the extent of themes and sub-themes without implying that ‘numbers reveal the truth in the data’.^38^

### Reporting of results

In keeping with the study’s sequential explanatory mixed-methods design, the results from the quantitative and qualitative phases are integrated under the major headings, namely, features of the online course that supported learning and challenges with the online course and improvements to the course.

### Ethical considerations

The Human Research Ethics Council (Medical) of the University of the Witwatersrand approved the study: Certificate Number M210938. Permission to conduct the study was obtained from the Faculty of Health Sciences Registrar and the Unit for Undergraduate Medical Education.

## Results

The response rate for the survey was 52% (*n* = 164/316), which approximated the estimated required response rate of 54%. The respondents were mainly black people (36.8%), female (66.5%) and in the age range 21 years to 25 years old (65.2%), which is closely representative of the general study population (60.4% female and 39.6% male) (Table 1).

**TABLE 1:** Respondent demographics.

Characteristic	Respondents (*n* = 164)		GEMP IV cohort (*N* = 316)		*p* *
	*n*	%	*n*	%	
**Gender**					
Male	54	33.5	124	39.24	0.197
Female	107	66.5	191	60.4	0.197
Other	3	1.83	1	0.32	-
Total	164	-	316	-	-
**Age (years)**					
21–25	107	65.2	222	70.3	-
≥ 26	57	34.8	94	29.7	0.299
Total	164	-	316	-	-
**Population group**					
Black people	60	36.8	126	39.9	0.410
White people	59	36.2	102	32.3	0.353
Indian people	26	16.0	67	21.2	0.199
Other	18	11.0	21	6.6	0.084
No response	1	-	-	-	-
Total	164	-	316	-	-

GEMP IV, Graduate Entry Medical Programme IV.

* , *p* < 0.05 indicates a statistically significant difference; *p* < 0.000 indicates a highly statistically significant difference.

Most respondents (93.3%) agreed that they could easily access the online course, with no significant difference (NSD) by gender (*p* = 0.341) or age (*p* = 0.418), and more than 80% of respondents indicated that support to address issues with the online course was readily accessible, with NSD by gender (*p* = 0.295) or age (*p* = 0.482).

### Features of the online course that supported learning

#### Statistical analysis

More than 80% of the survey respondents agreed that the course was logically structured, with NSD by age (*p* = 0.418) or gender (*p* = 0.295) and that the online activities helped structure and support their learning, with NSD by age (*p* = 0.945) or gender (*p* = 0.541). Sixty-four per cent of respondents felt the online content was relevant to managing patients in clinical settings, with NSD by gender (*p* = 0.059) or age (*p* = 0.373).

#### Content analysis

**FIGURE 1:**
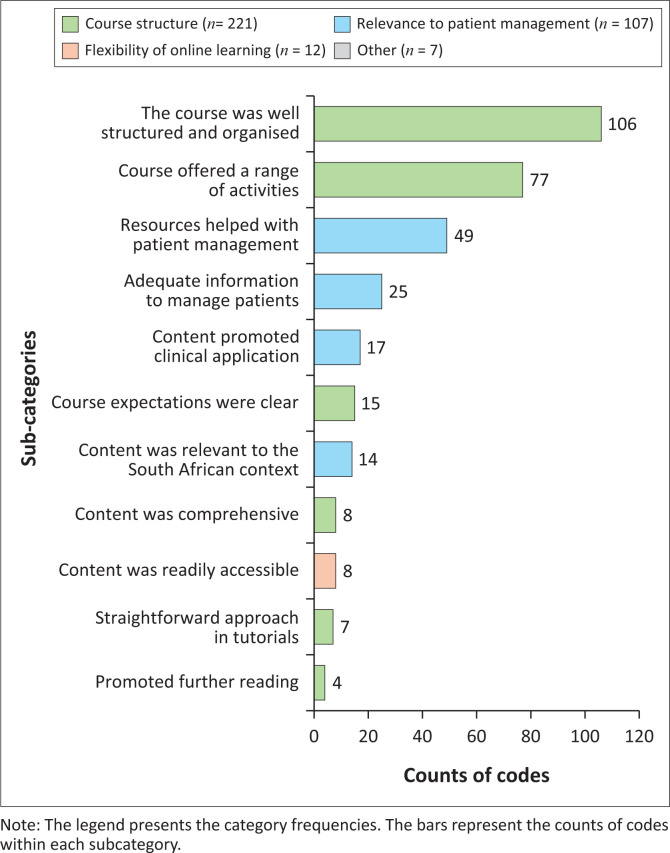
Features of the online course that supported learning (content analysis: *n* = 347; counts less than four are not shown).

The content analysis of the open-ended survey responses supported the statistical results that the ‘Course structure’ (*n* = 221/347) and the ‘Relevance to patient management’ (*n* = 107/347) were the leading categories of features that supported learning (Figure 1). The content analysis also reinforced that the main features of the course structure supporting learning were the course structure and organisation (*n* = 106) and the range of online activities offered (*n* = 77). The content analysis identified additional course features that supported learning, notably that the ‘course expectations were clear’ (*n* = 15/221) and the ‘content was comprehensive’ (*n* = 8/221). The respondents also valued the ‘straightforward approach in tutorials’ (*n* = 7/221) (Figure 1).

The second largest category of features that supported learning was related to the course’s ‘Relevance to patient management’ (*n* = 107/347). The main sub-categories were that the ‘resources helped with patient management’ (*n* = 49/107) and there was ‘adequate information to manage patients’ (*n* = 25/107). Respondents also stated that the course ‘content was relevant to the South African context’ (*n* = 14/107). The category ‘Flexibility of online learning’ (*n* = 12/342) was another aspect that supported student learning, with the main feature being the ready accessibility of the content.

#### Thematic analysis

**FIGURE 2:**
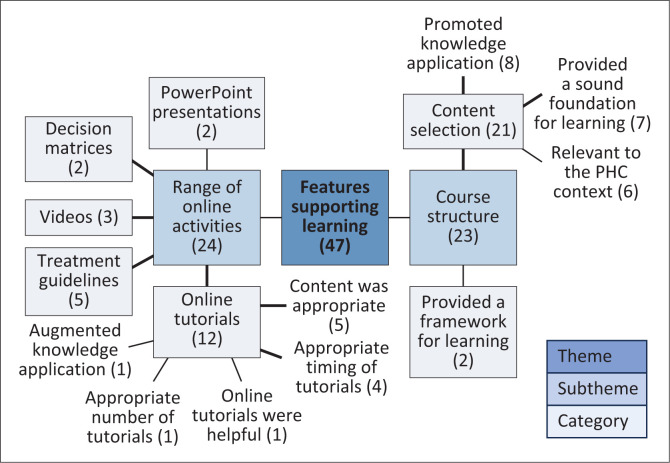
Thematic map showing features of the online course that supported learning.

The FGDs explored the findings from the content analysis more deeply. The first of the three themes identified from the thematic analysis, ‘Features supporting learning’, consisted of two sub-themes: the ‘range of online activities’ (*n* = 24) and the ‘course structure’ (*n* = 23) (Figure 2). The online tutorials (*n* = 12) featured prominently as an online activity that supported learning. The participants felt that the tutorials provided appropriate content (*n* = 4) and were appropriately timed (*n* = 4), confirming that the respondents felt that the tutorials covered common presenting conditions they were likely to encounter. The provision of ‘treatment guidelines’ (*n* = 5) was the second major online activity supporting learning. The following comment supports this finding:

‘Yes, it did contribute towards management. The reference to guidelines, I think, directly impacted my management of the patient, [*and*] the online content did support my understanding and the identification [*of the management plan*].’ (P2, FG1, female)

Regarding the ‘Course structure’, participants shared that the ‘content selection’ (*n* = 21) promoted knowledge application (*n* = 8), provided a sound foundation for learning (*n* = 7) and was relevant to the PHC context (*n* = 6) (Figure 2). The following quotes from Participant 4 from FGD 2 and Participant 2 from FGD 1 confirm the interpretation of the data.

‘… the content, I did find it useful. I found it more useful, actually, for the clinical part of the block, the actual being on the ground, seeing patients and because it did cover the conditions that we were seeing and the type of patients we were seeing.’ (P4, FG2, male)

‘I used additional content to study for it, and one of my mates [*laughs*] … he only used the online content and mainly focused on that and did a bit of reading around the, specifically, the online content. And ja, he actually did better than me. So, I think the online content is good. I think it’s sufficient.’ (P2, FG1, female)

The above-stated quote highlights that the perceptions of the purpose and value of online content may differ between students and educators. Students’ perception of the value of the online content may be based mainly on how they perform in assessments rather than on what they learn.

### Challenges with the online course

#### Content analysis

**FIGURE 3:**
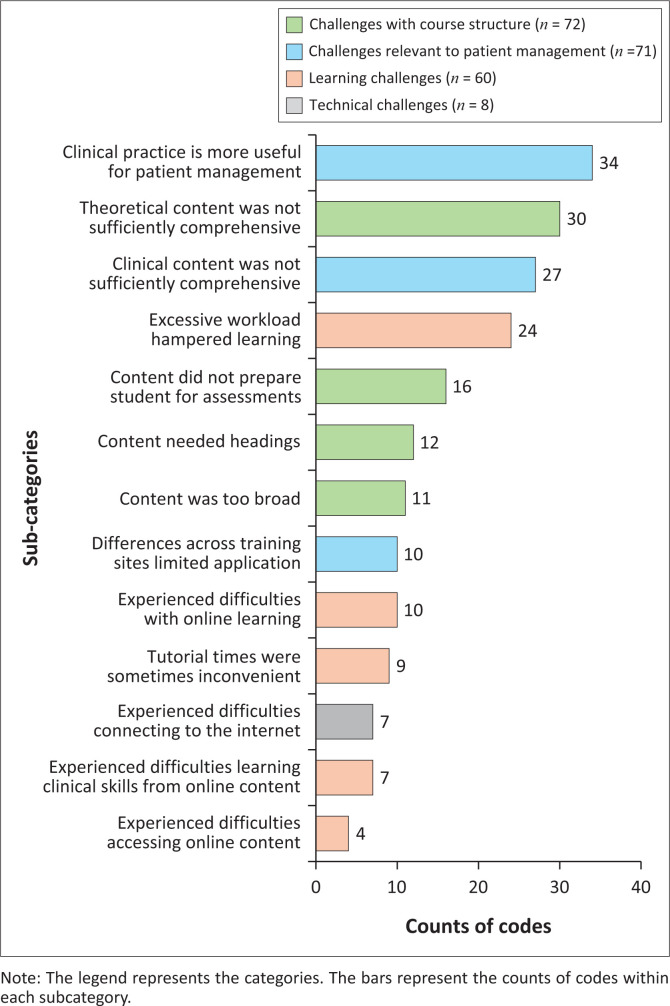
Challenges with the online course as identified from the content analysis (*n* = 211; counts less than four are not shown).

The content analysis identified four categories of challenges: the ‘Course structure’ (*n* = 72/211), ‘Challenges relevant to patient management’ (*n* = 71/211) ‘Learning challenges’ (*n* = 60/211) and ‘Technical challenges’ (*n* = 8/211) (Figure 3).

The major sub-categories of challenges were respondents’ perceptions that ‘clinical practice was more useful for patient management’ (*n* = 34/71) and that the ‘theoretical content was not sufficiently comprehensive’ (*n* = 30/72). Other notable sub-categories were that the ‘excessive workload hampered learning’ (*n* = 24/60), that respondents had ‘experienced difficulties with online learning’ (*n* = 10/60) and had also ‘experienced difficulties learning clinical skills from online content’ (*n* = 7/60).

#### Thematic analysis

**FIGURE 4:**
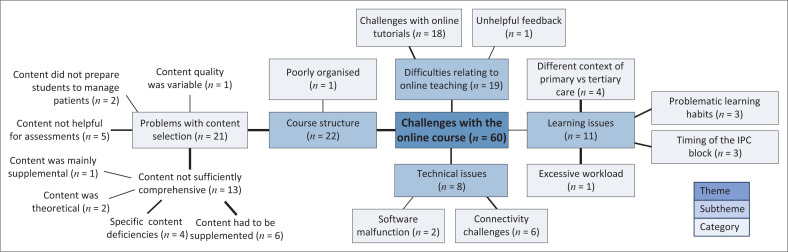
Thematic map showing the challenges with the online course.

The thematic analysis identified four sub-themes: ‘Course structure’ (*n* = 22), ‘Difficulties relating to online teaching’ (*n* = 19), ‘Learning issues’ (*n* = 11) and ‘Technical issues’ (*n* = 8) (Figure 4). The problems with the course structure were that the content was not sufficiently comprehensive (*n* = 13) and did not prepare them to manage patients (*n* = 2) or for assessments (*n* = 5). The participants also described challenges with the online tutorials (*n* = 18) and variability of content quality (*n* = 1) (Figure 4). One participant highlighted the inconsistency and difficulties of online teaching experienced by students:

‘Sometimes, you have a registrar who has been asked to do it [*the tutorial*] by their consultants, and they’re just not keen at all, which does impact on your learning.’ (P3, FG2, male)

The thematic analysis revealed additional learning challenges to those identified in the content analysis. The sub-theme, ‘Learning issues’ (*n* = 11), highlights the ‘different context of primary vs tertiary care’ (*n* = 4) as challenging for students. The thematic analysis also raised the issue of the ‘timing of the IPC block’ (*n* = 3) influencing knowledge integration, as explained by one of the participants:

‘We still have a lot of growing to do. It’s unbelievable how much growth we go through on a year-to-year basis. And so, if we haven’t got the benefits of those rotations under our belt, sometimes it can be a little bit difficult to know what … you know, what the objectives are, or what’s helpful to know.’ (P3, FG2, male)

As in the content analysis, the FGD participants described ‘connectivity challenges’ (*n* = 6) as the primary contributor to the technical challenges of learning online; see ‘Technical issues’ (*n* = 8) in Figure 4. A notable difference between the two types of analyses was that the FGD participants placed less emphasis on the excessive workload compared to the survey respondents.

### Improvements to the course

**FIGURE 5:**
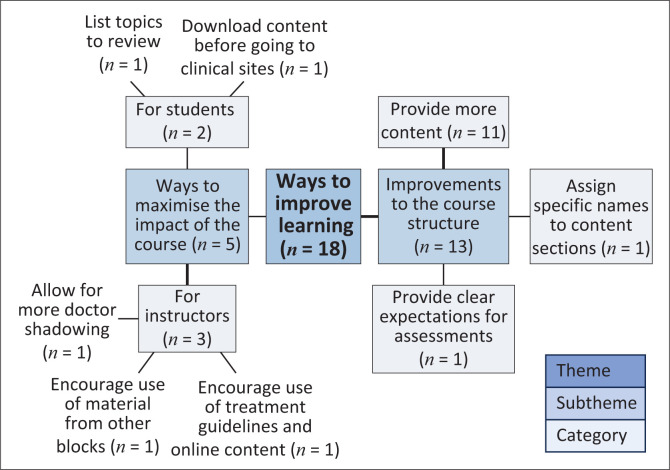
Thematic map of suggested improvements to the course.

The final theme identified from the thematic analysis consisted of participants’ suggestions to enhance the impact of the course, with two focal areas (Figure 5). One focus area centred on the structure of the online content (*n* = 13), with the need for more comprehensive content being the dominant recommendation (*n* = 11/13). One FGD participant justified the need for more content as follows:

‘It might be helpful to just supplement the online content a little bit more for those blocks that haven’t been … that students might not have covered yet, so that they just have more to … you know, like because they don’t have that information … that knowledge yet.’ (P4, FG2, male)

The other focus area suggested ways to maximise the impact of the course (*n* = 5) by making recommendations about how instructors (*n* = 3) and students (*n* = 2) use the course. For example, instructors could link the course content to content in other teaching blocks, encourage using the treatment guidelines provided in the course and allow for more doctor shadowing. One FGD participant explained:

‘We would really benefit from more shadowing of a doctor and working with the doctor in tandem and managing patients together. Being a team.’ (P3, FG2, male)

## Discussion

This explanatory-sequential mixed-methods study aimed to explore final-year medical students’ experiences and perceptions of online learning across DCT sites affiliated with Wits University during the COVID-19 pandemic in 2021. The results from an online survey (quantitative phase) informed FGDs conducted to gain insight into the survey results.

We identified a positive attitude towards using the online IPC course to supplement and augment learning during the COVID-19 pandemic. Positive attitudes to online learning have also been reported in several studies since the onset of the pandemic, for example, among dental students.^39^ A 2017 survey of medical students’ readiness for increased online learning at our institution also reported an overall positive attitude towards online learning.^40^ This survey, however, emphasised the negative impact of a lack of, among other factors, teacher and student preparedness for online teaching and learning.^40^

Course access is fundamental to course usage; therefore, it is critical to note that, in contrast to the Ingratta et al.^40^ pre-COVID-19 study, our survey respondents reported that it was easy to access the online course. Our analysis also did not identify any major problems with student access to devices or readiness to use them. These findings are critical, given the historical difficulties with internet access experienced in LMICs, partly because of the inequitable distribution of digital resources.^41^ The commitment from the university to assist students with access to devices and data to access the Internet may have mitigated some of the problems around access to the course. In response to the lockdown implemented during the pandemic, the university implemented a laptop programme and provided students and staff with 30GB of data per month. Despite the university’s interventions to facilitate access to online learning, long-term and widespread electricity instability afflicting the country during 2021 impacted internet access, in addition to technical issues and software malfunctions.

Holmberg^42^ points out that teaching is increasingly viewed as designing for learning, with sound instructional design contributing to effective course structure and organisation. The participants in this study predominantly reported that the structure of the online course enhanced their theoretical knowledge and clinical skills. They felt that the content selection and organisation, including clear learning objectives and the range of available activities, helped structure and focus their learning. These pedagogical elements are well supported in the literature. For example, Webb et al.^43^ acknowledged that providing clear learning objectives is essential for explicit course expectations, regarding these as more important than content organisation. Other notable design features that supported learning were the wide range of learning formats combined with traditional teaching and learning interactions. Numerous authors have noted that integrating online content with more conventional forms of clinical teaching positively affects medical education by providing flexibility, ease of distribution, relief of staff teaching burden and standardisation of content.^19^

A possible reason for the positive perceptions of the course design could be the deliberate teacher, social and cognitive presence built into it from the CoI model. The value of appropriate content selection and organisation with explicit learning objectives is underpinned by the teacher and cognitive presence of the CoI model.^23^

Despite the positive perceptions about the course, there were several challenges, one being the structure of the course. Suggestions to improve the content structure included adding headings or topic names, more supplementary resources and clear assessment expectations. The request for better structure indicates that perhaps the teacher presence of the CoI model, which has to do with the design of the course, was inconsistent or sub-optimal and can be improved. Another critical challenge was the online course’s relevance to clinically managing patients. One of the main objectives of designing the IPC online course during the COVID-19 pandemic was to augment patient management, acknowledging that clinical training is best taught at the bedside in clinical settings.^20^ Successful doctor shadowing has been noted as a valuable tool;^44^ therefore, the recommendation about including more doctor shadowing in IPC teaching could address the limitations of learning theoretical knowledge and clinical skills online. However, effective learning also requires active student participation.^45^ Active student participation could be encouraged by enhancing the social presence of the CoI model and promoting SRL by designing appropriate learning interactions.

Other challenges included the online content being regarded as incomplete and not preparing students for assessments. The idea of being inadequately prepared for assessments because of perceived incomplete content raises questions about whether we are training students to be successful test-takers or competent doctors. Assessment influences student learning, and perhaps educators need to rethink what and how we want to guide student learning to enrich their learning experience.^46^ Rethinking assessments using SRL and the social presence of the CoI model may be one way to deepen the learning process and encourage lifelong learning. It was perplexing that some respondents wanted more content despite the excessive workload being identified as a learning challenge. A possible reason for this could be the broadness of family medicine as a discipline,^47^ which warrants further reading on diverse subject topics. Another challenge was learning in PHC settings compared to more familiar tertiary care settings. Primary care is the focus of IPC, which suggests that perhaps more training is needed to equip students with the necessary clinical skills for the PHC setting and possibly to address the challenge of shortages of medical doctors in South African rural areas.

One notable recommendation from the study was that students prepare learning content before the start of the block to make the block more manageable. Preparing for the next block, which entails mainly organisational preparation rather than actual learning, is eminently possible because the learning content is available from the beginning of their final-year. Preparation aligns with the idea of forethought, in that preparation allows students to at least start to ascertain what knowledge and skills are needed to complete the IPC block successfully. Another suggestion focussed on more student participation in clinical teams, which clinical teachers could encourage by implementing more collaboration and sharing of clinical experiences via a more robust social presence in the online learning space. Incorporating the suggestions for clinical teachers to promote the use of the online content and guidelines, as well as content from other blocks, may also address the challenge of the timing of the IPC block. Drawing from other blocks could allow the IPC block to assist students in consolidating their knowledge.

Clinical teachers may need more professional development in designing more effective online interactions. Courses with clear learning objectives, a range of online activities, frequent tutorials, explicit instructions about assessments, and consideration of the training context could augment clinical learning. Additionally, clinical teachers would benefit from guidance on integrating online courses into their teaching.

### Limitations

The FGDs relied on non-probability sampling, which may have caused a self-selection bias and impacted the extent of the discussions in the focus groups. Self-reporting bias may result in measurement error, as there may be a deviation in measurement between self-reported and actual values. Additionally, this study relied on the interpretation of a single educator in one subject in one health profession at a South African university.

### Recommendations

- Health professions educators must be upskilled regarding effective blended and online teaching and learning pedagogies. The CoI model is an effective, efficient and authentic way to improve online learning.^23^ Ensuring a well-structured and organised online-learning platform with strong teacher, cognitive and social presence will assist with the development of a robust blended-learning approach. In addition, SRL, in blended and online learning with realistic expectations and goals, a grasp of the process during learning and self-reflection on the performance of the task once completed^48^ can be used to encourage learner participation in their own learning.
- Developing basic instructional design skills in health professions education may assist teachers in incorporating more digital tools to enhance student engagement and learning. Where teachers lack these skills or require support because of other factors, for example, workload issues, they should have the support of instructional designers to ensure the design of meaningful learning interactions.

## Conclusion

Overall, the participants displayed a positive attitude to learning online and the online learning content during their IPC block. The selection and organisation of content, range of online activities and clear learning objectives were largely appropriate. The challenges highlighted by the participants, such as the theoretical content not being sufficiently comprehensive and clinical practice being more useful for patient management, could be addressed by improvements in course design. The perception that the online content did not prepare students for assessments requires educators to rethink what and how we assess teaching outcomes to enrich learning. Design improvements may also address the challenges of excessive workload and learning difficulties. Finally, student inclusion in the clinical working environment could facilitate better use of content and support student learning.

This study highlights the importance of instructional design and the need for varied and ongoing faculty development to support teachers in their choice of content, pedagogy and online teaching tools. Let us not waste the opportunity to build on the lessons of COVID-19 in health professions education and work towards improved robust educational offerings.
